# Dissociating instructive from permissive roles of brain circuits with reversible neural activity manipulations

**DOI:** 10.1101/2023.05.11.540397

**Published:** 2025-09-08

**Authors:** Daniel Quintana, Hayley A. Bounds, Jennifer Brown, May Wang, J. Simon Wiegert, Hillel Adesnik

**Affiliations:** 1 Department of Neuroscience, University of California, Berkeley; 2 The Helen Wills Neuroscience Institute; 3 University Medical Center Hamburg-Eppendorf, Hamburg, Germany; 4 Department of Neurophysiology, MCTN, Medical Faculty Mannheim, Heidelberg University, Mannheim, Germany

## Abstract

Recent work has demonstrated that both permanent lesions and acute inactivation experiments can lead to erroneous conclusions about the causal role of brain areas in specific behaviors, casting serious doubt on major avenues by which neuroscientists study the brain. To overcome this challenge, we developed a three-stage optogenetic approach which leverages the ability to precisely control the temporal period of regional inactivation with either brief or sustained illumination, enabling investigators to dissociate between putative ‘permissive’ and ‘instructive’ roles of brain areas in behavior. We applied this approach to the mouse primary visual cortex (V1) to probe whether V1 is permissive or instructive for the detection low contrast stimuli. Acute inactivation of V1 drastically suppressed performance, but during persistent inactivation, the animals’ contrast detection recovered to pre-silencing levels. This recovery was itself reversible, as returning the animals to intermittent V1 inactivation reinstated the behavioral deficit. These results argue that V1 is the default circuit mice use to detect visual stimuli, but in its absence, other regions can compensate for it. This novel, temporally controllable optogenetic perturbation paradigm should be useful in other brain circuits to assess whether they are instructive or permissive in a brain function or behavior.

## INTRODUCTION

Neuroscientists rely on targeted perturbations to causally map functions in the brain^1^. Yet since the brain is highly interconnected, manipulation of one area can impact behavior through indirect effects on other brain regions, complicating the interpretation of such results^2,3^. Permanent lesions of one area can lead to dysfunction or degeneration of other brain regions, making it difficult to determine whether the functional consequences of the lesion are due to loss of the targeted region or to those that are indirectly damaged. Moreover, the often observed recovery of behavioral performance after a permanent lesion can further complicate the interpretation of lesion experiments: either the lesioned area was never directly involved, or another brain area was able to compensate for its loss^4,5^.

**Fig. 1.**
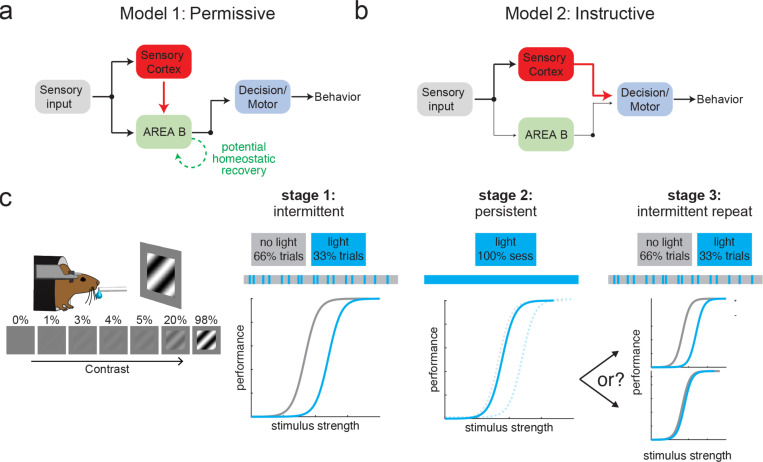
A three-stage optogenetic inactivation paradigm for dissociating instructive from permissive roles of a targeted brain circuit. **A)** A permissive model for the circuit. In this model, Area A is only needed to keep Area B in a functional state so that Area B instructs correct performance. When Area A is removed or permanently silenced (bottom), Area B can homeostatically recover (curved green arrow) from its loss of drive from Area A and restore correct behavior. **B)** An instructive model for the circuit. In this model, Area A directly instructs correct performance, but when it is removed or permanently silenced, a redundant accessory circuit through Area B restores performance (bottom). **C)** An approach to distinguish among these models with chronic, but reversible inactivation: three successive stages of intermittent, persistent, and repeat intermittent silencing of the targeted circuit. Bottom: schematic plots of behavioral performance on a relevant task showing performance in each stage for light off (gray) and light on (blue) trials. For stage 2, the dotted lines are the schematized performance data from stage one replotted for comparison. In this example set of results, in Stage 1 inactivation reduces performance, but in stage 2 performance recovers. In stage 3, intermittent inactivation either returns to reducing performance (top) or no longer has any effect (bottom).

Recent studies have highlighted how the results of acute and irreversible inactivation can conflict^4–6^. For example, acute inactivation of forebrain motor or sensory areas in animals can lead to profound behavioral deficits, but animals with permanent lesions are either unaffected or can recover performance within just a few days^4–6^. In one striking example, the behavioral deficit observed during acute inactivation of one forebrain region of the songbird could instead be attributed to an indirect effect on activity in a downstream area^4^. Once the downstream area homeostatically adjusted its activity, the animal’s behavior recovered. This led the authors to propose that a brain region can either be “instructive” – providing essential information that is used in the computation for a behavioral task, or “permissive” – acutely required, but not providing essential information or computation. A permissive region is only ‘required’ in that it might provide tonic excitatory or inhibitory tone to a downstream region to generally set that region’s gain or sensitivity (Fig. 1a). In song production, the forebrain region was merely permissive in the behavior rather than instructive.

Similarly, another study found conflicting effects of acutely optogenetically silencing or permanently lesioning the barrel cortex on performance in a whisker-dependent task. While acute silencing of barrel cortex impaired behavior, animals could entirely recover from an irreversible lesion of the barrel cortex within ~2 days, a process that was experience dependent^5^. This study offered two possible conclusions: either barrel cortex is instructive but is redundant with another area that quickly compensates for its loss^7^ (Fig. 1b); or barrel cortex is permissive and acute inactivation transiently disrupts the instructive area. Yet another study found that mice trained to discriminate pure tones recovered their performance following auditory cortex lesions in just a few hours, but did not recover performance during prolonged optogenetic silencing over the same timeframe, raising the possibility that lesions may uniquely engage compensatory mechanisms^6^. Together, these studies highlight that existing approaches for probing the roles of brain areas in behavior cannot easily resolve whether an area is instructive or permissive. Furthermore, when multiple brain areas can both be instructive in a task, they also cannot clearly determine which one might be the ‘default’ area the brain primarily relies on to solve it, and which is only secondary.

These studies demonstrate that acute manipulations alone can lead to erroneous or ambiguous conclusions about the instructive role of a brain area, such as the cortex, in a specific behavior. Yet acute, rapid, and reversible manipulations, such as with optogenetics^8–10^, have become central to the investigation of the brain and behavior. Compared to irreversible lesions, acute manipulations avoid potential indirect neurodegenerative effects^2,11^, permit rigorously interleaved control trials, and can address the timescales for when neural activity in specific regions is necessary for behavioral output^12,13^. However, the current inability to properly interpret the results of such acute manipulations is a major roadblock in neuroscience. Thus, we sought to develop a new paradigm that can help resolve the critical ambiguities associated with acute perturbations, providing crucial additional evidence to determine whether an area is permissive or instructive in a task, and if instructive, whether it is the default area that normally instructs the behavior.

## RESULTS

### A flexible optogenetic strategy for both acute and persistent silencing of neural circuits.

We reasoned that we could help address the ambiguities of existing perturbation approaches by employing a flexible optogenetic inactivation strategy in which one could acutely or chronically silence a brain area in the same animal. With such an approach, one could first measure the acute effects of inactivation of a brain area on a behavior, then chronically silence it to mimic an irreversible lesion, and finally return the brain area to acute inactivation (Fig. 1c). In cases where acute inactivation impairs behavior, animals might recover behavioral function during chronic optogenetic silencing, as they often do after irreversible lesions. In such cases, one might be able resolve the apparent conflict by finally returning to acute, intermittent silencing in the recovered animals. This last test could be critically informative: if recovered animals no longer showed any effect of acute silencing, this would suggest that the brain region only played a permissive role in the initial condition (Fig. 1c). Conversely, if a return to acute silencing in recovered animals reinstates the behavioral deficit, the brain region is more likely to directly contribute to the computational process in an instructive manner.

**Figure 2.**
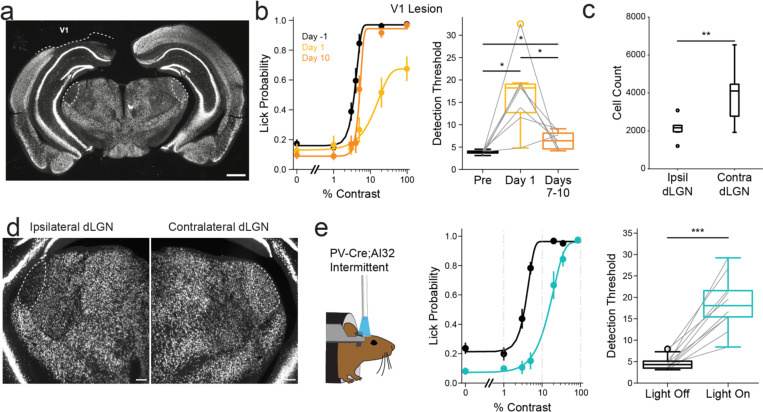
Conflicting effects of lesion and acute inactivation of V1 on behavioral performance in visual detection task **A)** Coronal section from a representative mouse stained with anti-NeuN, showing the extent of the V1 lesion. Dashed circles indicate the locations of the bilateral dLGNs. Scale bar: 1 mm. **B)** Left, psychometric performance curves for mice with V1 lesions measured on the day before lesioning (Day −1, black), the day after lesion (Day 1, yellow), and Day 10 post-lesion (orange). Lines represent Weibull function fits to the behavioral data. Right, boxplot showing detection thresholds averaged across six pre-lesion days, Day 1 post-lesion, and Days 7–10 post-lesion. Statistical comparisons: repeated-measures ANOVA, p < 0.05; paired t-tests with multiple comparison correction: Pre vs. Day 1: p < 0.05, Pre vs. Days 7–10: p < 0.05; Day 1 vs. Days 7–10: p < 0.05; n = 7 mice. **C)** Quantification of dLGN cell counts comparing ipsilateral (to the V1 lesion) and contralateral (to the V1 lesion) hemispheres (p < 0.01, paired t-test; n = 7 mice). **D)** Expanded view of the images of left (ipsilateral to the V1 lesion) and right (contralateral to the V1 lesion) dLGN from the section in (D). Scale bar: 100 μm. **E)** Left, psychometric performance curve for PV-Cre;Rosa-LSL-ChR2(Ai32) mice during intermittent silencing. Lines are Weibull function fits. Blue, light on, black, light off. Right, boxplot of detection thresholds during intermittent photostimulation; p<0.0001, paired t-test. n=10 mice.

To develop and leverage the proposed optogenetic strategy, we focused on the primary visual cortex (V1), which is among the best studied areas in the mammalian brain, but whose role in different aspects of visual perception remains in doubt. Prior work has shown that inactivating or lesioning V1 severely compromises visual function^3,12,14–16^, including in contrast sensitivity (particularly at higher spatial frequencies and/or small sizes)^17–20^, but that visual performance can recover spontaneously, with further training, or sometimes even with lesions to other brain areas^21–23^. We first confirmed these findings in mice trained on a visual contrast detection task^18,24^. We quantified performance using the detection threshold, or c50, estimated by fitting a psychometric function to the behavioral data. We found that permanently lesioning V1 resulted in an acute reduction in task performance, followed by a substantial, albeit incomplete recovery over the subsequent week (Fig. 2 a-b; Pre vs Day 1: p<0.05, Pre vs. Days 7–10: p<0.05, Day 1 vs Days 7–10: p<0.05. n=7 mice). To assess the extent to which surgery itself may have contributed to the observed deficits, we lesioned primary somatosensory cortex (S1) in a separate cohort of mice. Lesioning S1 had a dramatically smaller effect on visual behavior (Fig. S1 a-b), demonstrating that the initial deficit we observed following V1 lesion was specific and not due to surgery *per se* (Day 1 post lesion detection threshold, S1 lesion versus V1 lesion: p<0.05, n=10 mice). These data argue that following V1 lesion, mice exhibit substantial, but incomplete recovery of contrast sensitivity, raising two important questions: first, why does visual behavior recover at all? Behavior might recover because V1 is merely permissive and the brain homeostatically scaled up the gain of the instructive area. Alternatively, V1 is instructive, but behavior recovered because the animal learned to perform the task using alternative brain structures that are not normally required. Second, why is recovery incomplete? Either V1 is essential for a small component of behavioral performance, or because lesioning V1 leads to degeneration of other visual areas that are. Indeed, inspection of the primary visual thalamus in V1-lesioned mice showed a profound reduction in the number of thalamic neurons on the side ipsilateral to the lesion (Fig. 2c-d p<0.01, n=7 mice). Thus, based on these lesion results alone, we cannot conclude whether V1 is instructive or permissive in the contrast detection task, and we cannot rule out that distal degeneration of other visual areas is the source of lasting the behavioral deficit. These data highlight key problems with interpreting the results of conventional lesioning studies.

To begin to address these challenges, we next sought to use a reversible perturbation to clarify the role of V1 in basic visual functions in mice as other studies have done ^18,19,25^. We silenced excitatory neurons by optogenetically stimulating GABAergic neurons which caused a profound reduction in contrast sensitivity on illumination trials (Fig. 2e; PV-Cre; Rosa-LSL-ChR2(Ai32): p<0.0001, n=10 mice). Similar illumination in mice not expressing any opsin did not affect performance (Fig. S1c, p = 0.86, paired t-test, n=4 mice), controlling for any direct effects of the illumination on behavior. At face value, this acute inactivation experiment supports an instructive role of V1 in contrast detection, but it seemingly conflicts with the substantial recovery seen in mice with V1 entirely removed (Fig. 2b). Taken together, the results of irreversible and acute, reversible lesions of V1 highlight the difficulty of drawing definitive conclusions about its role in behavior even with a combination of approaches.

**Figure 3.**
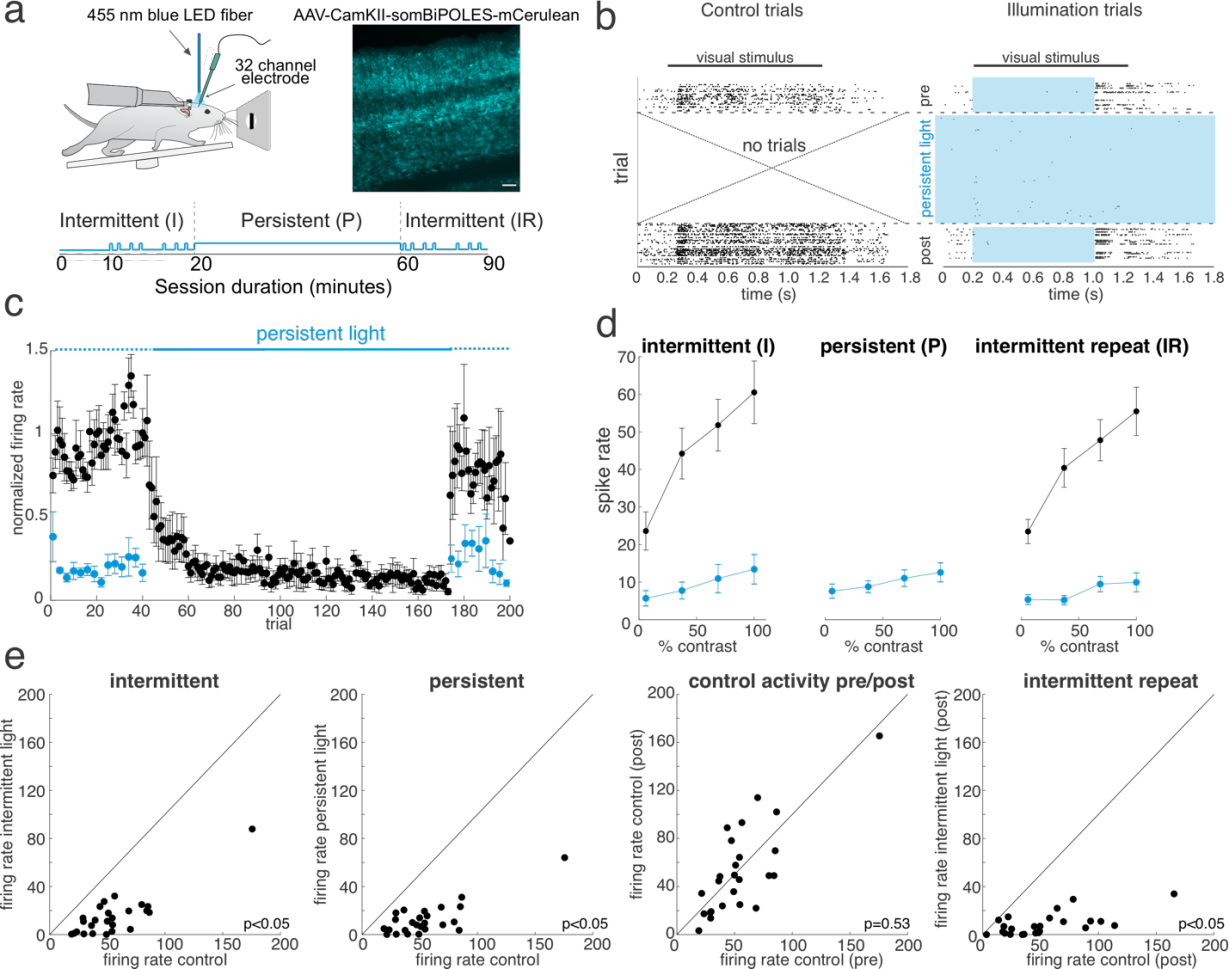
Acute and persistent optogenetic suppression of cortical activity. **A)** Left: schematic of the recording configuration. Right: Example image of a post-mortem slice of visual cortex expressing the inhibitory opsin throughout the cortical layers. Scale bar: 100 μm. Bottom: schematic of the illumination procedure with an intermittent illumination block, followed by 40 minutes of continuous persistent illumination, followed by a return to intermittent illumination. **B)** Example raster plot for all spikes on an example electrode during the acute/persistent optogenetic suppression protocol. Left: trials with no illumination during the intermittent phases. Right: trials during the illumination phases (blue rectangle); intermittent (pre), persistent, and intermittent (post), as indicated by the blue shaded regions. **C)** Averaged normalized spiking activity across all recorded tetrodes across all experiments (3 mice, 6 sessions, 25 tetrodes) during each phase of the optogenetic protocol for the 100% contrast stimulus. Intermittent illumination for ~40 trials, followed by continuous illumination for ~130 trials, followed by a return to intermittent illumination. Black dots correspond to no light trials, blue dots indicate illumination trials. Each phase of illumination is schematized by the blue bars above the plot; broken lines: intermittent; solid bar: persistent light. **D)** Average contrast response plots across all recorded units in each phase of the silencing experiment. Left: control (black) and intermittent illumination trials (blue). Middle: persistent illumination trials. Right: return to the intermittent illumination. **E)** Scatter plots of recording unit firing rates between the indicated conditions; 3 mice, 6 sessions, 25 tetrodes; Wilcoxon sign rank test. All error bars are s.e.m.

While optogenetic manipulations have conventionally been used to silence brain regions or cell types only briefly, we reasoned that prolonged optogenetic silencing might add crucial information by mimicking a permanent lesion, but ultimately still being reversible. For this approach we employed direct optogenetic silencing of cortical excitatory neurons by expressing the blue light silencer stGtaCR2 via the construct somBiPOLES^26^. To test the efficacy of silencing neural activity with BiPOLES under both intermittent and persistent illumination conditions, we recorded neural activity with multielectrode arrays across the layers of V1 while illuminating the cortex briefly on every third trial, and then persistently for 40 continuous minutes (2mW/mm^2^, 455 nm) (Fig. 3a-c). In BiPOLES-expressing mice, brief illumination resulted in immediate and strong suppression of both spontaneous and visually evoked neural activity (Fig. 3c-e; 83±4% reduction at 100% contrast, p<0.05, Wilcoxon signed rank test). Next, we asked if uninterrupted sustained illumination could effectively silence cortical activity for the entire duration of the illumination, and whether neural activity would rapidly recover after cessation of illumination to rule out potential photo-toxic effects. Indeed, during persistent illumination, the mean firing rate averaged over all recorded units was suppressed by 76±4% (Fig. 3d, e; at 100% contrast, p<0.05, Wilcoxon sign-rank test). After the cessation of sustained illumination, the mean activity was indistinguishable from baseline levels (Fig. 3e; p=0.53, Wilcoxon signed rank test), arguing against potential issues with prolonged illumination^26^ at this light level, 2mW/mm^2^, or that prolonged flux of ionic currents might lead to cellular alterations^27^. These experiments confirm the efficacy, stability, and reversibility of these acute and more persistent optogenetic manipulations.

### Acute and persistent effects of V1 inactivation in a visual detection task

**Fig. 4.**
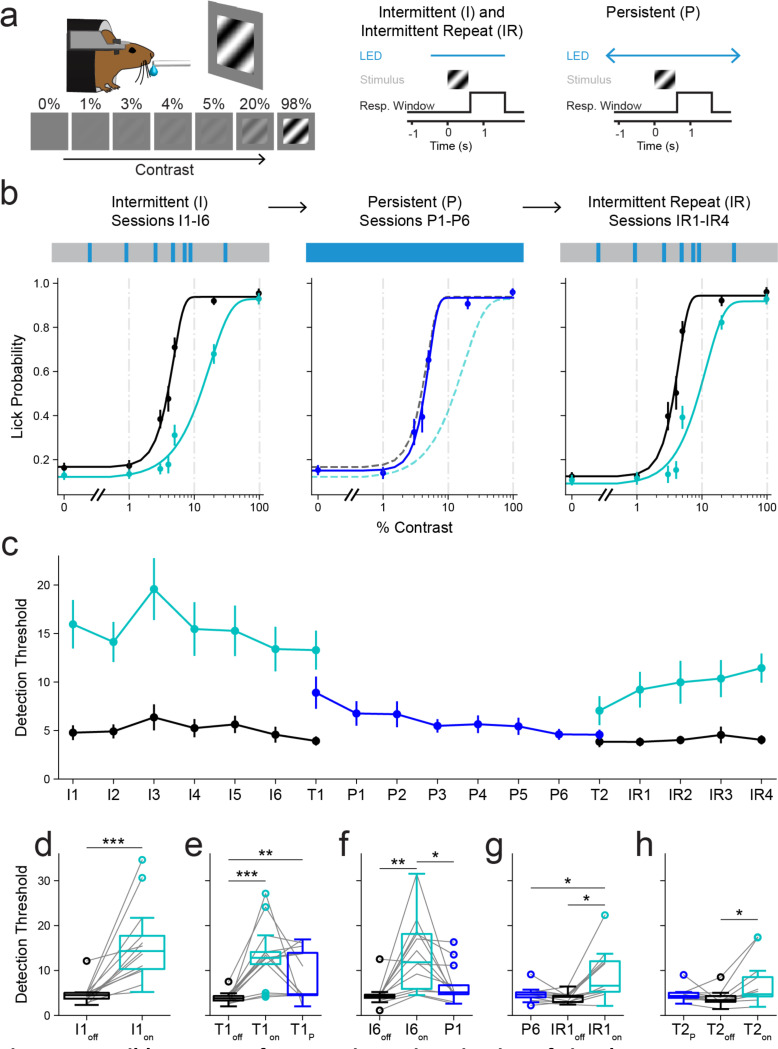
Reversible recovery from persistent inactivation of visual cortex. **A)** Left: Schematic of visual detection task. Right: Optogenetic trial structure: Light is on transiently (~2 seconds) for 30% of trials (intermittent and intermittent repeat, left) or chronically (persistent, right) for the duration of the session. Gray bar represents stimulus presentation time, black line represents response window, time referenced to the start of the stimulus window. **B)** Top, Schematic of silencing blocks, excluding transition days. In the intermittent blocks, silencing was applied on a subset of trials. In the persistent block, silencing was continuous throughout each session. The intermittent repeat block had the same structure as the intermittent block. Bottom, psychometric curves of performance for n = 10 mice tested at the most common contrast set. Left, intermittent block, light blue shows light on performance and black is light off performance. Center, persistent block, performance during silencing shown in dark blue, with dashed lines indicating intermittent performance replotted from the left panel for comparison. Right, intermittent repeat block, colors as in intermittent. **C)** Detection threshold in each day of the testing protocol. Transition days (T1 & T2) had one half of the session intermittent, one half persistent. n=13 mice. **D)** Detection threshold in light off (black) vs light on (blue) trials for intermittent day 1 (I1) (p<0.001, n=13 mice, paired t-test). **E)** Detection threshold for T1. In the first half of the session intermittent silencing occurred on light on (light blue) trials interspersed within light off (black) trials. In the second half light was turned on for the entire period for persistent silencing (dark blue). p<0.001, repeated measures ANOVA (rmANOVA). Pairwise comparisons: I_on_ vs I_off_: p<0.001, I_on_ vs P: p=0.08, I_off_ vs P: p<0.01; multiple-comparisons corrected paired t-test; n=13 mice. **F)** Detection threshold during light off vs light on trials for intermittent day 6 (I6) vs persistent day 1 (P1). P<0.001, rmANOVA. Pairwise comparisons: I6_on_ vs P1: p=0.12, I6_on_ vs I6_off_: p<0.001, I_off_ vs P: p<0.01, multiple-comparisons corrected paired t-test; n=13 mice. **G)** Detection thresholds in persistent day 6 (P6) versus intermittent silencing day 1 (IR1). p<0.01 rmANOVA. IR1_on_ vs P6: p<0.05, IR1_on_ vs IR1_off_: p<0.05, IR1_off_ vs P6: p=0.15, multiple-comparisons corrected paired t-test; n=11 mice. **H)** Detection thresholds for T2, which is the inverse of T1 (e). p<0.01 rmANOVA. IR_on_ vs P: p=0.18, IR_on_ vs IR_off_: p<0.05, IR_off_ vs P: p=0.24; n=13 mice. B-C) Data are shown as group mean ± s.e.m. D-H) Boxplots show the median (center line), interquartile range (box limits: 25th to 75th percentile), and whiskers (extending to the most extreme data points within 1.5× the interquartile range). Outliers beyond the whiskers are shown as individual points.

Equipped with this flexible optogenetic approach, we probed how acute versus persistent optogenetic inactivation of V1 would impact performance on the task. We trained somBiPOLES-expressing mice on the same visual contrast detection task used above^18,24^ (Fig. 3a) and quantified performance using the c50, or detection threshold, as above. Consistent with results above in which we silenced the cortex by stimulating PV neurons, intermittent silencing excitatory neurons severely impaired performance (Fig. 4b, left; Fig. S2: p<0.001; paired t-test, n=13 mice).

To better mimic a permanent lesion, we then illuminated V1 for the entire duration of each behavioral session. Notably, this avoids irreversibly lesioning the tissue so that silencing can be switched off in future sessions. Moreover, it avoids silencing V1 outside the task, such as in the home cage, which might on its own cause local or downstream circuit reorganizations^30–33^. We applied this persistent illumination scheme to the same mice as above to test if mice might recover performance over time despite continued suppression of V1. Between the last day of intermittent silencing and the first day of persistent silencing, we included a ‘transition day’ in which the first half of the session employed intermittent silencing and the second half employed persistent silencing to look for fast changes in the role of V1 in behavior. After this day, the optogenetic light was on for the entirety of each session for six consecutive days (session duration: 57±13 minutes; mean ± SD). On average, performance during the persistent silencing was not significantly different from light on performance in the intermittent portion (Fig. 4e; Transition Day 1 (T1); Intermittent light on (I_on_) vs Persistent: p=0.12, I_on_ vs I_off_: p<0.001, I_off_ vs P: p<0.01; n=13 mice). However, on the first full day of persistent silencing (‘P1’), the mice substantially recovered performance on the task such that their detection threshold under persistent illumination was not significantly different from their performance without illumination on the last full day of intermittent silencing (Fig. 4f-h; Intermediate silencing day 6 (I6) vs persistent day 1 (P1); I6_on_ vs P1: p<0.05, I6_on_ vs I6_off_: p<0.01, I6_off_ vs P1: p=0.12; n=13 mice). These data demonstrate that mice can recover much of their contrast sensitivity even in the continued suppression of V1 activity. Moreover, the very rapid recovery that we observed on the first day of persistent silencing imply that mice can rapidly switch to using alternative circuits to detect contrast. This, along with the recovery observed after mechanical lesion, suggests that normal V1 activity is not absolutely required for task performance. Thus, so far, the data leave it unclear whether V1 is instructive or permissive in the task.

### Reversion to a reliance on V1 activity after cessation of persistent silencing.

The data above show that the mice can adapt to solve a contrast detection task when V1 is very strongly suppressed or even entirely ablated by exploiting alternative brain circuits. Thus, V1 is not required for task performance, but may still be instructive in the normal condition. We reasoned that we could address this key question by reverting the persistently silenced and behaviorally recovered mice to intermittent silencing. If intermittent silencing of V1 no longer had a behavioral effect, this would imply that the cortex is probably not the default circuit instructing performance on this visual task and is likely merely permissive. Conversely, if mice instead return to failing on intermittent V1 silencing trials, this will imply that V1 is the default circuit that actively instructs perceptual behavior.

To discriminate between these possibilities, we reverted the persistently silenced animals to intermittent silencing the cortex only on 33% of trials, as before (intermittent repeat, IR). To resolve any fast changes in behavioral performance, we started with a second transition day composed of persistent silencing in the first half and intermittent silencing in the second half. Then we began the intermittent repeat block of four sessions of intermittent silencing alone. Surprisingly, we found that during the first day of intermittent repeat (IR1) block, on average mice returned to showing a dramatic reduction in performance during intermittent silencing trials (Fig. 4b,c,g IR1_on_ vs P: p<0.05, IR_on_ vs IR_off_: p<0.05, I_off_ vs P: p=0.15; n=11 mice). By the end of the intermittent repeat sessions (IR4), the performance in intermittent silencing trials was the same before as in at the end of the original intermittent silencing block (I6), before the behavioral recovery during persistent illumination (Fig S3a; p=0.44, paired t-test, n=13 mice). This reversion to requiring V1 activity is consistent with V1 playing an instructive rather than a merely permissive role in the visual task.

Again, to address how fast this reversion to relying on V1 activity might occur, we examined the transition session between the P and IR blocks. During the intermittent half of the session, there was already a significant difference between light off and light on (Fig 4h; Transition Day 2 (T2); Pairwise comparisons: IR_on_ vs P: p=0.18, IR_on_ vs IR_off_: p<0.05, IR_off_ vs P: p=0.24; n=13 mice). These findings emphasize how quickly mice go back to relying on V1 activity. More generally, these results with a return to intermittent silencing demonstrate that the behavioral recovery observed during persistent inactivation of V1 is itself reversible: once the animal regains access to normal V1 activity it goes back to relying on it. This further implies that V1 is instructive, rather than permissive.

Another advantage of our flexible optogenetic paradigm is that V1 has normal activity between each behavioral session, which can help further disambiguate between instructive and permissive roles. These ‘off’ periods, where V1 activity is unperturbed, might allow potential homeostatic adjustments in other brain areas to revert between sessions. To test this, we carefully analyzed performance across persistent silencing sessions. If homeostatic recovery of other brain areas explains behavioral recovery during V1 silencing, we would expect that it would likewise revert between persistent silencing sessions (when V1 activity is normal). In this case, performance should be worse at the start of each persistent silencing session and recover by its end. However, hit rates in the first fifth of a persistent session were comparable to those at the end of the prior session (Fig. S3b,c, p=0.82, n=13 mice), suggesting that whatever recovery occurs, it does not occur repeatedly within each session. This provides additional evidence against a homeostatic explanation for the behavioral recovery during persistent silencing, implying instead that the mice learn to use an auxiliary brain area to detect visual stimuli in the absence of V1 activity.

## DISCUSSION

Here, we present a simple and potentially broadly applicable three stage paradigm for using optogenetics to help disambiguate whether a given brain circuit is instructive or permissive in a behavior. In stage one, acute inactivation assesses whether the target circuit is involved at all but does not discriminate between an instructive or permissive role. In stage two, sustained inactivation assesses whether the behavior can recover, either due to relearning with alternative circuits or due to homeostasis. Finally, when there is behavioral recovery, stage three returns to acute inactivation to help determine whether the targeted circuit is instructive or permissive.

Our data on mouse V1 supports a model in which V1 is instructive in perceptual behavior and not merely permissive. It is the result from the return to intermittent inactivation that best distinguishes between the multiple competing explanations for why acute V1 suppression impairs behavior, but persistent silencing yields recovery. First, the performance recovery could have been explained by homeostatic recovery of a downstream area (e.g., the superior colliculus) whose normal activity level depends on sensory cortical input. If this were true, then when the mice were reverted to intermittent silencing, one would have expected the mice to be resistant to cortical inactivation since the downstream area had homeostatically become independent of cortical drive. However, this was not the case as mice quickly reverted to depending on V1 activity. It is possible that the homeostatic recovery could itself have disappeared in the intervening time between the last persistent inactivation session and the first intermittent inactivation session of the subsequent block. However, if this were true it should have happened between each of the persistent inactivation sessions, and then mice should have shown transient poor performance at the beginning of each persistent inactivation session. However, this was not the case (Fig. S3c), arguing against any spontaneous recovery between days of persistent illumination.

In a second scenario, the recovery of performance during persistent silencing could have been explained by the animals permanently relearning the task using alternative circuits that do not depend on primary sensory cortical activity^5^. In this scenario, task relevant information is available to the animal in both the primary sensory cortex and alternative structures independently, and though originally the animal depended on V1 activity to solve the task, over several days of training in its absence, the animal would have relearned the task via these alternate circuits. In this scheme, again one would have expected that the animals would be resistant to reversion to intermittent inactivation of V1 since they had learned to solve the task independent of the primary sensory cortex. However, this was not the case, as again, animals very quickly returned to showing poor performance upon reversion to intermittent silencing (Fig. 4b,c,g,h). This does not support the second explanation.

In a third scenario, V1 is part of the default circuit that instructs the animal’s downstream decision circuits to solve the perceptual task. When V1 is inactivated intermittently, the animal fails on the task since it is relying on primary sensory cortical output for instructing performance. When V1 activity is unavailable for long enough, the downstream decision circuits eventually adapt to employing an alternate source of information to solve the task that may always have been present but was originally ineffective at driving these circuits. This learned reliance on alternate circuits is, unlike in scenario two, impermanent: when primary sensory cortical activity again becomes available, the downstream circuits revert to ignoring this alternative source of information even on trials in which the primary sensory cortex is acutely inactivated. Our results support this third scenario; they argue that V1 is the default source of instructive information for solving a simple contrast detection task.

Taken together, this new multi-stage optogenetic approach can generally help address the challenges posed by potential off-target effects of acute circuit perturbations. In principle, any study already using optogenetic inactivation can execute this paradigm without substantial additional technical complexity, although it is important to confirm the efficacy of sustained optogenetic suppression. The main advantage is that using this paradigm should lead to much more interpretable results. This should obviate the need to exhaustively search for potential alternative neural pathways with which an animal can solve a task (although such a search would still be valuable). Moreover, it may aid in understanding the mechanisms of behavioral recovery after brain lesions^28^, helping to delineate the default versus accessory circuits that can contribute to specific behaviors.

## MATERIALS AND METHODS

### Animals

Mice used for experiments in this study were C57/BL6J (Jax stock# 000664) and PV-IRES-Cre (Jax stock# 008069) crossed with Rosa-LSL-ChR2 (PV-Cre;Rosa-LSL-ChR2(Ai32)) (Jax stock# 012569)^35,36^. Mice were housed in cohorts of five or fewer in a reverse light:dark cycle of 12:12 hours, with experiments occurring during the dark phase. Both female and male mice were used. For optogenetic silencing with somBiPOLES, a total of C57/BL6 13 mice were used (7 males) aged 10 – 20 weeks. For the V1 silencing in Figure 1 10 PV-Cre; Ai32 mice were used (5 males) aged 8 – 15 weeks. For lesion experiments, a total of 11 C57/BL6 mice were used (3 males) aged 12 – 30 weeks.

### Viral injection

Neonatal C57/BL6 mice (P3-P5) were cryoanesthetized and their head was placed in a ceramic mold and secured with non-abrasive tape. Viral solutions (AAV9-CaMKII-somBiPOLES-mCerulean, Addgene) were loaded into beveled glass injection needles (WPI) and mounted in a NanoJect II (Drummond). The needle was inserted through the skin and scalp over the left V1 (2.3 mm lateral and 0.1 mm anterior to lambda) using a micromanipulator (Sutter MP-285) at three locations, each spaced approximately 0.3 mm apart. Virus was injected at four depths within V1 (400, 300, 200, and 100 μm), with 20 nl of viral solution delivered per site at a rate of 10 nl/second.

### Headpost surgery

All experiments were performed in accordance with the guidelines and regulations of the Animal Care and Use Committee of the University of California, Berkeley. For head fixation during behavioral and physiological experiments, a small custom stainless-steel headplate was surgically implanted. Briefly, adult mice (P70 – P 155) were anesthetized with 2–3% isoflurane and mounted in a stereotaxic apparatus. Buprenorphine (0.05–0.1 mg/kg SC) was administered for analgesia and dexamethasone (2 mg/kg SC) to prevent cerebral edema. Body temperature was monitored and maintained at 37°C. The scalp was removed, the fascia retracted, and the skull lightly scored with a drill bit. Vetbond (3M) was applied to the skull surface, and the headplate was fixed to the skull with Metabond (C&B). A fine-point marker was used to note the approximate location of the left primary visual cortex (V1; 2.7mm lateral, 0mm posterior to lambda). For all behavioral experiments involving mice injected with somBipoles, a glass cranial window was implanted over the marked site to provide optical access to the cortex. In V1 silencing comparison experiments, the skull was instead thinned and covered with a single layer of Vetbond to enhance light penetration. Similarly, for electrophysiological recordings, the skull was thinned to transparency on the day of the experiment to ensure optimal electrode access and signal quality. Mice received buprenorphine and meloxicam for pain management and could recover for at least three days before being placed on water restriction.

### Lesion surgery

Water restricted adult mice (P75-P180) previously headplated (see headpost surgery for procedure) and trained to perform the behavioral paradigm were anesthetized using isoflurane 2.5% and mounted in a stereotaxic apparatus. Buprenorphine (0.05–0.1 mg/kg SC) was administered at anesthetic induction. The eyes of the animal are covered with ocular lubricant to keep eyes moist. A dental burr was used to perform a 3 mm × 4 mm rectangular craniotomy over the left visual cortex (V1; 2.7mm lateral, 0mm posterior to lambda). Exposed cortical tissue was aspirated using a vacuum-attached blunt needle (16–30 awg). The lesion cavity is extended ventrally until the alveus surface of the hippocampus is exposed. The cavity was covered and sealed using a glass coverslip. Animal received meloxicam and buprenorphine post-surgery for analgesia. Mice performed on the behavioral paradigm 2–4 hours post-surgery and performance is reported as lesion day.

### In vivo electrophysiology recordings

Mice were anesthetized with isoflurane (2.5%), and a small craniotomy (<250 microns) was opened over V1 with a fine needle. Mice were transferred to the electrophysiology rig where they were head fixed and allowed to wake and run freely on a circular treadmill. A 32-channel multielectrode array (NeuroNexus, A1×32–5mm-25–177-a32) was guided into the brain using a micromanipulator (Sutter Instruments) and a stereo microscope (Leica). Electrical activity was amplified and digitized at 30kHz (spikeGadgets Inc.) and stored on a computer hard drive. The bottom of the electrode was zeroed with the surface of the brain to determine z depth. The electrode array was lowered ~650–750 microns into V1. A multimode fiber (400 μm diameter) was positioned ~2–3mm from brain next to the recording electrode to deliver 455 nm LED light during optogenetic illumination at a total of 2mW/mm^2^ measured at the tip of the fiber.

Spike detection was performed across assigned tetrodes (four adjacent electrode contacts) using SpikeGadgets threshold detection algorithm. Analysis was performed on all detected spikes on each tetrode. Not all assigned tetrodes were used because the top tetrodes were often located in L1 or outside of the brain. Statistically significant differences between conditions were determined using the Wilcoxon sign rank test across all tetrodes. Since mCerulean was not readily visible during adult surgeries, all experiments were initially performed blind to successful opsin expression, and animals with no obvious physiological optogenetic effects (strong activation by red light via the Chrimson in somBiPOLES), were excluded, which correlated with weak, or no expression observed post-mortem.

### Water restriction

Initial animal weight was recorded for 3 days before water restriction to establish baseline weight. Mice were then placed water restriction. On training days, mice received most water during the task. Mice were weighed after training and given additional water if their weight dropped below 70% initial weight. Food was available *ad libitum*. The weight and health (quality of the fur and nails, gait, posture) of the mice were monitored daily.

### Behavioral training on the contrast detection task:

#### Apparatus

For the contrast detection task, the visual behavioral apparatus was controlled in real time using an Arduino DUE (A000067) synchronized with Raspberry Pi 3+/4+ (BCM2837B0/SC0193(9)) and a custom software in Python, Arduino and Java^29^. The mouse was head fixed bilaterally and placed on a 3D-printed holder tube. Mouse licking was measured through the detection of changes in the capacitive load in a touch sensor (Adafruit, AT42QT1070) caused by the contact of the mouse’s tongue and a 0.05-inch diameter steel lickport. Water was delivered through the lickport using a 2-way normally closed isolation valve control (Neptune Research Inc.). Visual stimuli were displayed in a gamma corrected LCD monitor (Podofo, 15.7 × 8.9 cm, 1024×600 pixels, 60 Hz refresh rate) positioned 12 cm from the right eye with a 30-degree angle from the longitudinal axis.

#### Behavioral training and task design

Head fixed mice were trained to lick when they detected a visual stimulus of variable contrasts displayed on the monitor. The training started after weight dropped below 80% of the initial weight from water restriction. Prior to training, mice were habituated to head-fixation for two daily sessions lasting 30 minutes each. The behavioral training protocol consisted of four stages.

In stage 1, mice were classically conditioned to lick in response to a visual stimulus that consisted in a square patch with drifting sinusoidal grating (2Hz, 0.08 cycles/degree, 600 ms, 100% contrast, 34 visual degrees, black background luminescence). Each visual stimulus was paired with a water reward delivered at the beginning of a 1000 ms response window. Mice who achieved a performance of more than 90% of correct trials were transitioned to the next phase.

In stage 2, mice were operantly conditioned to respond every time the same stimulus from stage 1 was displayed (2Hz, 0.08 cycles/degree, 600 ms, 100% contrast, 34 visual degrees, black background luminescence). Mice received a water reward when they licked within a 1000 ms response window after the visual stimulus presentation. A no-lick period was enforced after two seconds of the trial. Catch trials were introduced and consisted of 25% total trials. Mice licking in a no-lick period, or a catch trial received a 5 to 9 seconds time out punishment. Mice achieving performance with a hit rate >90% and false alarm rate <30% for two consecutive sessions were switched to the next phase.

In stage 3, mice continued to train in the same behavioral paradigm with increased background luminescence at different sessions until it reached mean luminescence. Then, the visual stimulus size was decreased in different sessions until it reached 18 VD. The same performance criteria as in stage 2 was used in every session before transitioning to any change.

In stage 4, mice started to detect stimuli of six different contrasts, pseudo-randomly distributed. The intensities were customized for each mouse to provide a reliable detection threshold. Mice were introduced to the optogenetic protocol once performance was stable for more than 3 sessions. During photostimulation experiments, catch trials were 25% of all trials and response window was reduced to 500 ms. On average, mice completed 375 trials per session on non-transition days, and 420 trials for the entire session on transition days.

### Optogenetic stimulation during behavior:

For V1 one-photon optogenetic experiments, an optical fiber (400 μm diameter, 0.39 nA; M98L01, Thorlabs Inc.) coupled to a 455 nm LED (M4553, Thorlabs) and attached to a LED driver (LEDD1B, Thorlabs), was placed above the mouse’s window or skull thinned area using a micromanipulator (MM3, Narishige). Light power was calibrated daily and measured from the tip end of the fiber using a power meter (PM160T, Thorlabs). Photostimulation power was set to 2.0 mW for somBiPOLES-injected mice, 5.0 mW for non-opsin-expressing controls, and ranged from 2.0–8.0 mW for PV-Cre;Ai32 mice. During the intermittent blocks, optogenetic stimulation were 33% of all trials, pseudo-randomly distributed in blocks. Light was delivered at a random time prior to the stimulus onset, between 300 – 3000 ms, to avoid cueing the mouse to the trial start. Light was sustained until the end of the response window. During the persistent block, optogenetic stimulation was turned on prior to the beginning of the session and remained on until the session ended. All som-BiPOLES mice underwent 14 consecutively consisting of 6 sessions each I, P and IR blocks, with two additional transition days (T1 and T2) between different blocks. However, only 4 IR sessions were included in the analysis, as some mice were inadvertently exposed to incorrect contrast intensities during the final 2 sessions of this stage. A custom designed removable light blocking system was attached to the head of the mouse to prevent the mice from seeing the optogenetic stimulation light. A masking light and a plastic eye patch over the left eye were also placed to prevent additional extraneous cues.

### Histology

Mice were anesthetized with 5% isoflurane and ketamine in preparation for transcardial perfusion with phosphate-buffered saline (PBS), followed by 4% paraformaldehyde (PFA). Brains were removed, post-fixed overnight in PFA at 4°C, rinsed three times in PBS, then cryoprotected in 30% sucrose for 1–2 days. Brains were monitored to ensure they initially floated and subsequently sank, indicating complete cryoprotection. Afterward, a sliding microtome (American Optical Company), frozen using dry ice and ethanol to ensure proper adhesion and sectioning, was used to obtain 40 μm coronal sections of S1 and/or V1.

For NeuN staining, sections were incubated for 1 hour at 4°C on a rocker in blocking solution containing 3% normal goat serum (NGS), 0.6% Triton X-100, 0.2% Tween-20, and 3% bovine serum albumin (BSA) in PBS. Primary antibody incubation was performed overnight at 4°C using Rabbit anti-NeuN (Abcam) diluted 1:10,000 in blocking buffer. The following day, sections were washed in PBS with 0.25% Triton X-100 (PBS-T) and incubated for 1 hour at room temperature with Alexa Fluor 405–conjugated Goat anti-Rabbit IgG H&L secondary antibody (1:1000; Thermo Fisher Scientific). Sections were washed again in PBS-T, mounted onto glass microscope slides, and allowed to dry before applying coverslips and Vectashield mounting medium (Vector Laboratories) to reduce movement artifacts.

Imaging was conducted using an Olympus MVX10 Stereo Microscope with a 2× and 0.63x objective lens, 1.8× zoom, 0.5 numerical aperture, and 500 ms exposure. Cell counting was performed using a custom QuPath v0.2.3 pipeline automated for detection of NeuN-positive cells within manually defined regions of interest across sequential brain slices, using intensity thresholds and cell segmentation to quantify labeled nuclei.

### Quantification and Statistical Analysis

Hit rate (% Hit) was defined as the number of trials go trials (contrast > 0) where the mouse correctly licked within the response window divided by the total number of go trials. Days where mice were run on the incorrect contrast levels due to experimenter error were excluded. For detection threshold analysis, data for individual sessions were fit using a Weibull cumulative distribution function that fit the false alarm (lower asymptote), lapse rate (upper asymptote), slope and threshold parameter. Functions were fit separately for photostimulation and no-photostimulation trials. Detection threshold was defined as the contrast at which the fit hit ratewas halfway between the fit false alarm (lower asymptote) and fit lapse rate (upper asymptote).

Statistical analyses were performed using MATLAB or python. The analyses performed were repeated measures analysis of variance (rmANOVAs) with spherical correction when sphericity was violated, paired t-tests with Holm-Bonferroni corrections where needed, independent t-tests, and Wilcoxon rank sign tests. Unless otherwise noted, all tests were two-tailed and all plots with error bars were reported as mean ± SEM, all boxplots show the median (center line), interquartile range (box limits: 25th to 75th percentile), and whiskers (extending to the most extreme data points within 1.5× the interquartile range). Outliers beyond the whiskers are shown as individual points. Sample size was not predetermined using power analysis.

## Supplementary Material

Supplement 1

## Data Availability

All behavioral and electrophysiological data will be made available upon publication.

## References

[1] Lomber S. G. The advantages and limitations of permanent or reversible deactivation techniques in the assessment of neural function. J Neurosci Methods 86, 109–117 (1999). 10065980 10.1016/s0165-0270(98)00160-5

[2] Carrera E. & Tononi G. Diaschisis: past, present, future. Brain 137, 2408–2422 (2014). 24871646 10.1093/brain/awu101

[3] Lashley K. S. Mass action in cerebral function. Science 73, 245–254 (1931). 17755301 10.1126/science.73.1888.245

[4] Otchy T. M. Acute off-target effects of neural circuit manipulations. Nature 528, 358–363 (2015). 26649821 10.1038/nature16442

[5] Hong Y. K., Lacefield C. O., Rodgers C. C. & Bruno R. M. Sensation, movement and learning in the absence of barrel cortex. Nature vol. 561 542–546 Preprint at 10.1038/s41586-018-0527-y (2018). 30224746 PMC6173956

[6] O’sullivan C., Weible A. P. & Wehr M. Auditory cortex contributes to discrimination of pure tones. eNeuro 6, (2019). 10.1523/ENEURO.0340-19.2019 PMC6795560 31591138

[7] Cohen J. D. & Castro-Alamancos M. A. Early sensory pathways for detection of fearful conditioned stimuli: tectal and thalamic relays. J Neurosci 27, 7762–7776 (2007). 17634370 10.1523/JNEUROSCI.1124-07.2007 PMC3881290

[8] Fenno L., Yizhar O. & Deisseroth K. The development and application of optogenetics. Annu. Rev. Neurosci. 34, 389–412 (2011). 21692661 10.1146/annurev-neuro-061010-113817 PMC6699620

[9] Boyden E. S., Zhang F., Bamberg E., Nagel G. & Deisseroth K. Millisecond-timescale, genetically targeted optical control of neural activity. Nat. Neurosci. 8, 1263–1268 (2005). 16116447 10.1038/nn1525

[10] Nagel G. Channelrhodopsin-2, a directly light-gated cation-selective membrane channel. Proc Natl Acad Sci U S A 100, 13940–13945 (2003). 14615590 10.1073/pnas.1936192100 PMC283525

[11] Cowey A., Alexander I. & Stoerig P. Transneuronal retrograde degeneration of retinal ganglion cells and optic tract in hemianopic monkeys and humans. Brain 134, 2149–2157 (2011). 21705429 10.1093/brain/awr125

[12] Zatka-Haas P., Steinmetz N. A., Carandini M. & Harris K. D. Sensory coding and the causal impact of mouse cortex in a visual decision. Elife 10, (2021). 10.7554/eLife.63163 PMC8324299 34328419

[13] Resulaj A., Ruediger S., Olsen S. R. & Scanziani M. First spikes in visual cortex enable perceptual discrimination. Elife 7, (2018). 10.7554/eLife.34044 PMC5902162 29659352

[14] Petruno S. K., Clark R. E. & Reinagel P. Evidence That Primary Visual Cortex Is Required for Image, Orientation, and Motion Discrimination by Rats. Plos One 8, (2013). 10.1371/journal.pone.0056543 PMC3575509 23441202

[15] Burgess C. P. High-Yield Methods for Accurate Two-Alternative Visual Psychophysics in Head-Fixed Mice. Cell Rep. 20, 2513–2524 (2017). 28877482 10.1016/j.celrep.2017.08.047 PMC5603732

[16] Schneider G. E. Two visual systems. Science 163, 895–902 (1969). 5763873 10.1126/science.163.3870.895

[17] Prusky G. T. & Douglas R. M. Characterization of mouse cortical spatial vision. Vision Res 44, 3411–3418 (2004). 15536009 10.1016/j.visres.2004.09.001

[18] Glickfeld L. L., Histed M. H. & Maunsell J. H. R. Mouse Primary Visual Cortex Is Used to Detect Both Orientation and Contrast Changes. Journal of Neuroscience 33, 19416–19422 (2013). 24336708 10.1523/JNEUROSCI.3560-13.2013 PMC3858618

[19] Ruediger S. & Scanziani M. Learning speed and detection sensitivity controlled by distinct cortico-fugal neurons in visual cortex. Elife 9, (2020). 10.7554/eLife.59247 PMC7748414 33284107

[20] Del Rosario J. Lateral inhibition in V1 controls neural and perceptual contrast sensitivity. Nat. Neurosci. 28, 836–847 (2025). 40033123 10.1038/s41593-025-01888-4 PMC12743187

[21] Dean P. Visual acuity in hooded rats: effects of superior collicular or posterior neocortical lesions. Brain Res 156, 17–31 (1978). 568019 10.1016/0006-8993(78)90076-8

[22] Sabel B. A. & Kasten E. Restoration of vision by training of residual functions. Curr Opin Ophthalmol 11, 430–436 (2000). 11141637 10.1097/00055735-200012000-00008

[23] Sprague J. M. Interaction of cortex and superior colliculus in mediation of visually guided behavior in the cat. Science 153, 1544–1547 (1966). 5917786 10.1126/science.153.3743.1544

[24] Histed M. H., Carvalho L. A. & Maunsell J. H. R. Psychophysical measurement of contrast sensitivity in the behaving mouse. J. Neurophysiol. 107, 758–765 (2012). 22049334 10.1152/jn.00609.2011 PMC3289478

[25] Cone J. J., Scantlen M. D., Histed M. H. & Maunsell J. H. R. Different Inhibitory Interneuron Cell Classes Make Distinct Contributions to Visual Contrast Perception. Eneuro 6, (2019). 10.1523/ENEURO.0337-18.2019 PMC6414440 30868104

[26] Owen S. F., Liu M. H. & Kreitzer A. C. Thermal constraints on in vivo optogenetic manipulations. Nat. Neurosci. (2019) doi:10.1038/s41593-019-0422-3. PMC6592769 31209378

[27] Mahn M., Prigge M., Ron S., Levy R. & Yizhar O. Biophysical constraints of optogenetic inhibition at presynaptic terminals. Nat. Neurosci. 19, 554–556 (2016). 26950004 10.1038/nn.4266 PMC4926958

[28] Krakauer J. W., Carmichael S. T., Corbett D. & Wittenberg G. F. Getting neurorehabilitation right: what can be learned from animal models? Neurorehabil Neural Repair 26, 923–931 (2012). 22466792 10.1177/1545968312440745 PMC4554531

[35] Hippenmeyer S. A developmental switch in the response of DRG neurons to ETS transcription factor signaling. PLoS Biol. 3, e159 (2005). 15836427 10.1371/journal.pbio.0030159 PMC1084331

[36] Madisen L. A toolbox of Cre-dependent optogenetic transgenic mice for light-induced activation and silencing. Nat. Neurosci. 15, 793–802 (2012). 22446880 10.1038/nn.3078 PMC3337962

