# Supplementary material for: Dissociating instructive from permissive roles of brain circuits with reversible neural activity manipulations
Source: bioRxiv. 2025 Sep 8:2023.05.11.540397. Originally published 2023 May 12. Preprint. [Version 2] doi: 10.1101/2023.05.11.540397 (PMC10197619; doi:10.1101/2023.05.11.540397)
Supplement: Supplement 1 [file NIHPP2023.05.11.540397v2-supplement-1.pdf]

bioRxiv preprint doi: https://doi.org/10.1101/2023.05.11.540397; this version posted September 8, 2025. The copyright holder for this preprint (which was not certified by peer review) is the author/funder, who has granted bioRxiv a license to display the preprint in perpetuity. It is made available under aCC-BY 4.0 International license.

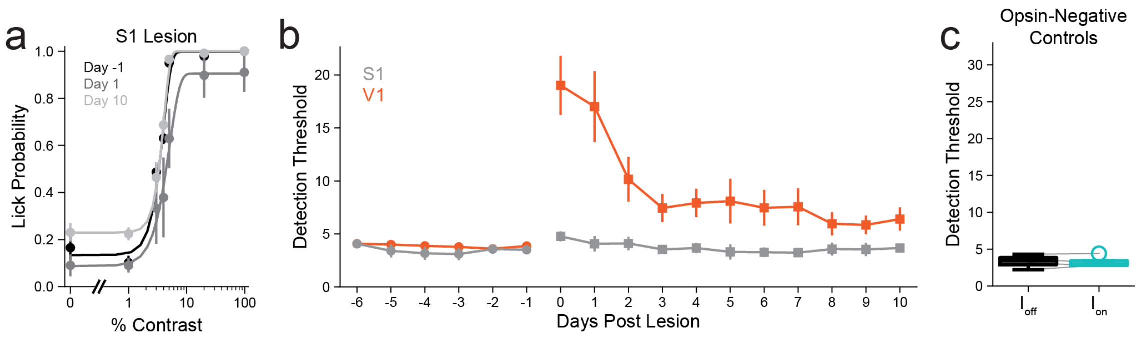

Fig S1. Control experiments for the specificity of V1 lesion and optogenetic inactivation on contrast detection

A) Psychometric curves of performance of mice with S1 lesions on the day immediately prior to lesion (Day -1, black), day after lesion (Day 1, dark gray), and Day 10 (light gray) after lesion. Lines, Weibull function fits of the psychometric curve.

B) Performance of mice for six days before lesion (circles), tested hours after lesion (Day 0), and then tested for ten days after lesion (squares). Orange, V1 lesion, n=7 mice, gray, S1 lesion, n=3 mice.

C) Boxplot showing detection thresholds in opsin negative controls averaged 3 sessions per mouse (p = 0.85, paired t-test, n=4 mice).

bioRxiv preprint doi: https://doi.org/10.1101/2023.05.11.540397; this version posted September 8, 2025. The copyright holder for this preprint (which was not certified by peer review) is the author/funder, who has granted bioRxiv a license to display the preprint in perpetuity. It is made available under aCC-BY 4.0 International license.

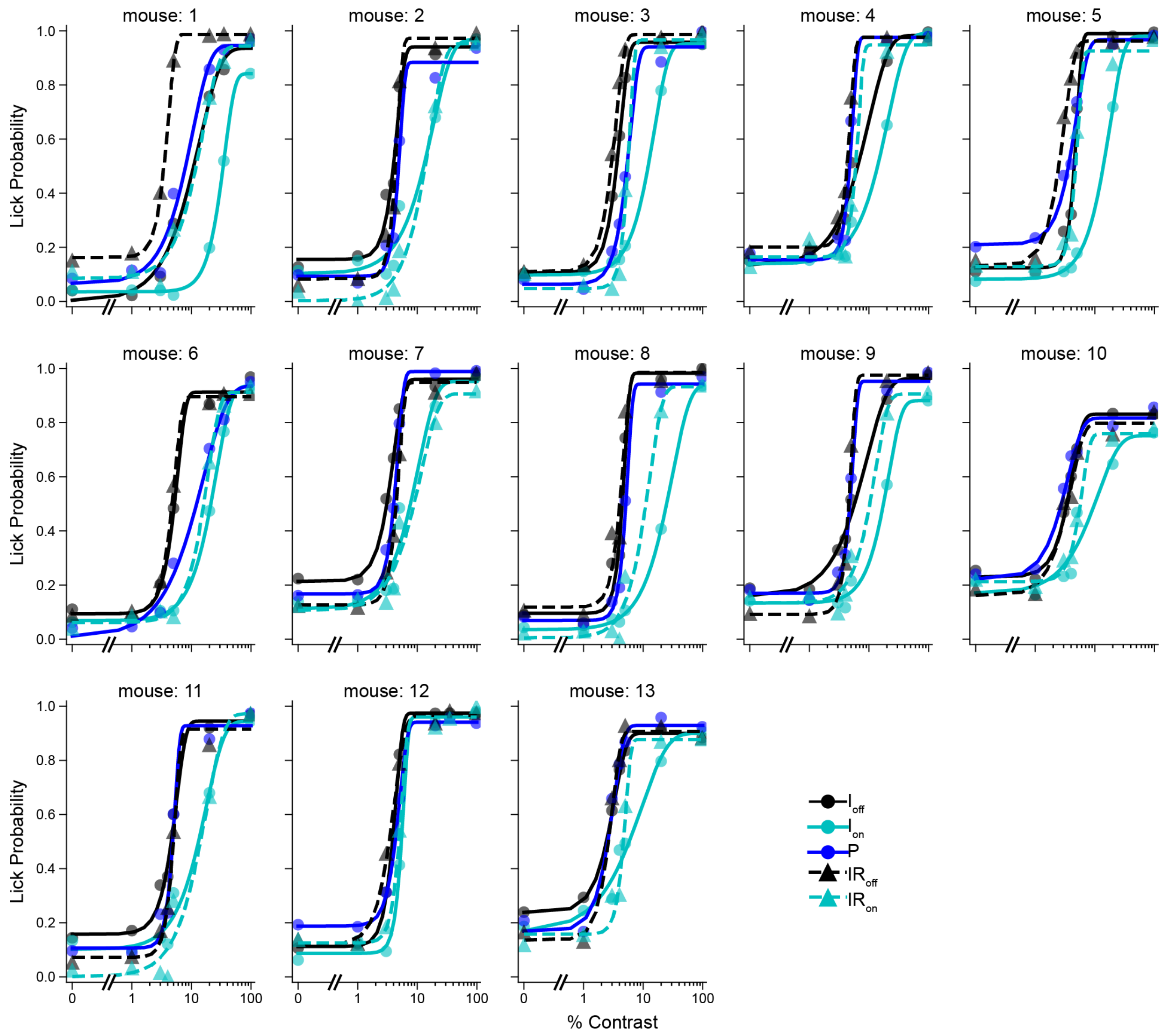

Fig. S2. Behavioral performance curves for each mouse. Psychometric curves of performance for each mouse during each stage of the task, fit with a Weibull function. Data are group mean ± s.e.m.

bioRxiv preprint doi: https://doi.org/10.1101/2023.05.11.540397; this version posted September 8, 2025. The copyright holder for this preprint (which was not certified by peer review) is the author/funder, who has granted bioRxiv a license to display the preprint in perpetuity. It is made available under aCC-BY 4.0 International license.

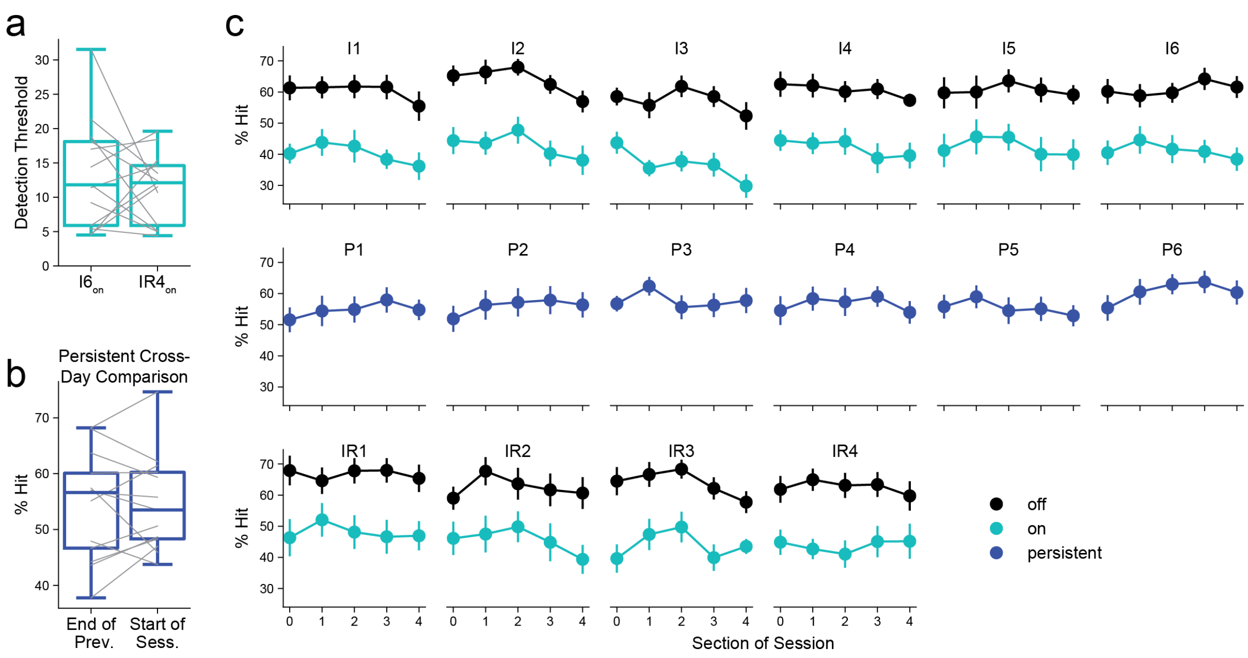

Fig. S3. Intermittent versus intermittent repeat and within-session performance comparisons.
A) Comparison of light on detection thresholds for intermittent day 6 (I6) and intermittent repeat day 4 (IR4) p=0.44, paired t-test, n=13 mice**.**
B) Comparison of, for persistent silencing days, last bin of performance on prior days vs bin on next day (Fig. S3b,c, p=0.82, paired t- test; n=13 mice).
C) Binned performance over each block (top: intermittent, middle: persistent, bottom: intermittent repeat) and for each session (1 to 6; left to right) for no light (black) and light (color) trials. Group mean ± s.e.m.
